# Classic Protocadherin PCDH10 Functions as a Tumor Suppressive Scaffold Protein Antagonizing Oncogenic WNT/β-catenin Signaling in Breast Carcinogenesis

**DOI:** 10.7150/ijbs.127857

**Published:** 2026-01-08

**Authors:** Xiaoyu Wang, Yiqing Tan, Yuanyuan Wang, Lili Li, Tingxiu Xiang, Yongheng Chen, Weiyan Peng, Zhu Qiu, Hongzhong Li, Guosheng Ren, Qian Tao

**Affiliations:** 1 Department of Breast and Thyroid Surgery, Chongqing Key Laboratory of Molecular Oncology and Epigenetics, The First Affiliated Hospital of Chongqing Medical University, Chongqing, China.; 2 Department of Breast Surgery, Sichuan Provincial People's Hospital, School of Medicine, University of Electronic Science and Technology of China, Chengdu, China.; 3 Cancer Epigenetics Laboratory, Department of Clinical Oncology, State Key Laboratory of Translational Oncology, Sir YK Pao Center for Cancer, The Chinese University of Hong Kong, Hong Kong SAR, China.

**Keywords:** PCDH10, tumor suppressor gene, methylation, breast cancer, WNT signaling

## Abstract

Epigenetic mechanisms, including DNA methylation, frequently inactivate tumor suppressor genes (TSGs) in multiple tumorigeneses. This study investigated the molecular basis of the tumor-suppressive role of the classic protocadherin tumor suppressor *PCDH10* in breast carcinogenesis. Frequent *PCDH10* downregulation and promoter methylation was identified in breast cancer, correlating with poor prognosis and ER-negative status. Restoration of *PCDH10* expression significantly suppressed tumorigenesis both *in vitro* and *in vivo,* by inhibiting epithelial-mesenchymal transition (EMT) and cancer stemness. RNA sequencing revealed *PCDH10*'s role in Wnt/β-catenin signaling suppression. Mechanistically, PCDH10 enhanced GSK-3β phosphorylation at Try216, inhibited aberrant β-catenin activation and upregulated the expression of the tumor-suppressive nuclear envelope protein LMNA expression through direct binding. Concurrently, it also attenuated other oncogenic signaling *via* suppression of RhoA and Akt phosphorylation. Collectively, promoter CpG methylation-mediated silencing of *PCDH10* promotes breast cancer progression. *PCDH10* restoration antagonizes tumorigenesis by dual blockade of Wnt/β-catenin and Akt signaling pathways through interactions with GSK-3β, β-catenin, and LMNA, as a scaffold protein. Our findings reveal a novel *PCDH10*-dependent tumor-suppressive axis and highlight its potential as a therapeutic target and biomarker in breast cancer.

## Introduction

Breast cancer remains the most common malignancy and second leading cause of cancer-related mortality among women worldwide 1. While diagnostic methods and adjuvant therapies have advanced considerably 2, the molecular pathogenesis of breast cancer remains incompletely understood. Carcinogenesis is a multi-step process that involves accumulated multiple epigenetic and genetic alterations 3, with epigenetic silencing of tumor suppressor genes (*TSG*) playing a critical role. Aberrant methylation of CpG islands (CGI) in TSG promoters represents a predominant epigenetic inactivation mechanism, triggering recruitment of repressive chromatin complexes and transcriptional silencing 4. Notable TSGs silenced by promoter methylation in breast cancer include *RASA5* 5, *HOXA5* 6, *DLEC1* 7, *RASSF1A* 8, and *ZDHHC1* 9, underscoring the significance of epigenetic dysregulation in breast carcinogenesis. Nevertheless, further mechanistic insights are required for breast cancer study.

PCDH10, a member of the protocadherin subfamily within the cadherin superfamily, regulates cell-cell adhesion and signaling 10-12. Our prior work identified *PCDH10* as an epigenetically silenced novel TSG, as the first protocadherin gene with repressive cancer-associated promoter hypermethylation, in multiple cancers 13, 14. Later, it was found that *PCDH10* suppresses tumorigenesis through modulating oncogenic pathways such as EGFR/AKT in colorectal cancer 15, and PI3K/AKT in hepatocellular carcinoma 16. In breast cancer, *PCDH10* promoter methylation occurs frequently and shows promise as a diagnostic biomarker 17, 18. However, its functional consequences, clinical relevance, and molecular mechanistic contributions to breast cancer pathogenesis remain poorly defined.

Thus, this study comprehensively investigates the clinical and functional significance of *PCDH10* in breast carcinogenesis. We delineate its tumor-suppressive mechanisms, evaluate the clinical relevance of its promoter methylation in disease progression, and dissect its functional interplay with core oncogenic pathways.

## Results

### *PCDH10* is frequently downregulated and methylated in breast cancer

**Figure 1:**
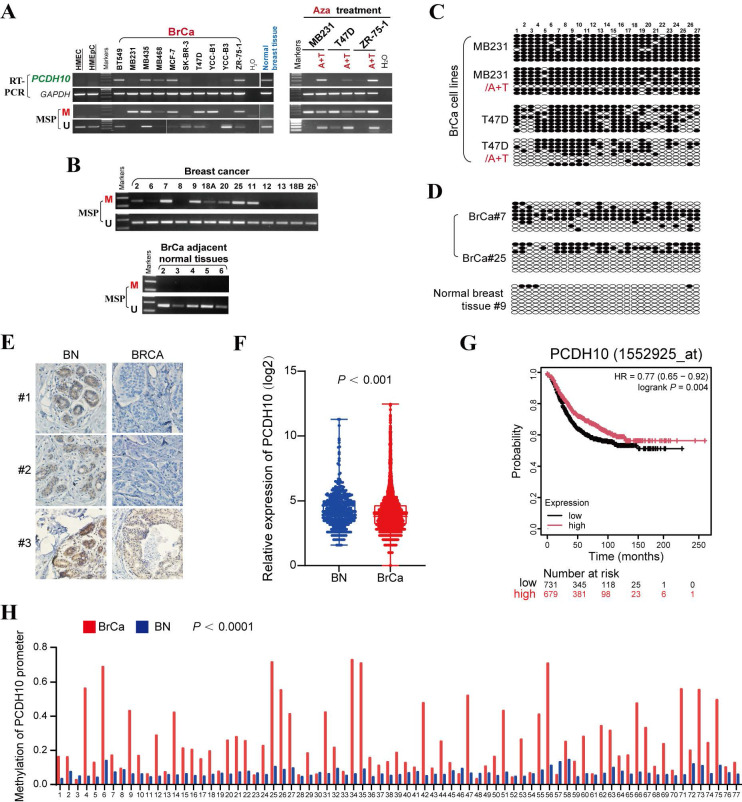
***PCDH10* is downregulated by promoter methylation in breast cancer. A** *PCDH10* mRNA expression level was detected by RT-PCR in breast cancer cell lines and normal breast tissues. *PCDH10* promoter methylation status was detected by MSP. mRNA expression and promoter methylation were further detected by RT-PCR and MSP in MDA-MB-231, T-47D, and ZR-75-1 with Aza and TSA treatment. A: Aza. T: TSA. **B** Representative MSP results of *PCDH10* in breast cancer and adjacent normal breast tissue samples. M, methylated; U: unmethylated. **C** BGS showing high-resolution mapping of methylation status of every CpG site within *PCDH10* promoter in A+T-treated MDA-MB-231 and T-47D. **D** BGS determining methylation status of every CpG site in representative breast cancer and normal breast samples. **E** IHC staining of breast cancer and normal breast tissues. **F** Online database GENT2 was used to examine *PCDH10* expression in normal breast and breast cancer. **G** Effect of *PCDH10* expression on survival in breast cancer patients based on Kaplan-Meier. **H** Analysis of *PCDH10* promoter methylation status in breast cancer and normal breast based on METHC. RT-PCR: semi-quantitative (RT)-PCR; MSP: Methylation-specific PCR; BrCa: breast cancer; BN: normal breast.

To investigate the epigenetic regulation of *PCDH10* in breast cancer, *PCDH10* mRNA expression was first analyzed. Semi-quantitative RT-PCR revealed that *PCDH10* transcript levels were reduced or silenced in 6/10 (60%) breast cancer cell lines, whereas robust expression was observed in normal breast tissue (Fig. 1A). Subsequent promoter methylation analysis by methylation-specific PCR (MSP) demonstrated *PCDH10* promoter methylation in 6/10 (60%) cell lines with absent or reduced expression (Fig. 1A), suggesting a strong correlation between *PCDH10* promoter methylation and its transcriptional silencing. To further investigate whether promoter methylation is directly responsible for *PCDH10* silencing, breast cell lines MDA-MB-231, T-47D, and ZR-75-1 were treated with 5-aza-2'-deoxycytidine (Aza), a DNA methyltransferase inhibitor, and Trichostatin A (TSA), a histone deacetylase inhibitor. This combined treatment aims to reactivate epigenetically silenced genes by reversing DNA hypermethylation and promoting a transcriptionally permissive chromatin state. Notably, the treatment dramatically restored *PCDH10* expression, while MSP analyses showed a concomitant decrease in methylated alleles and an increase in unmethylated alleles (Fig. 1A). Clinical validation studies revealed *PCDH10* promoter methylation in 43/52 (83%) primary breast tumor samples, but not in adjacent non-tumor tissues (0/5) (Fig. 1B, Table 1). Furthermore, bisulfite genomic sequencing (BGS) confirmed dense CpG island methylation in representative tumors (cases 7 and 25), in contrast to minimal methylation in a normal tissue sample (case 9) (Fig. 1C, D). Consistent with the observed transcriptional repression in tumors, immunohistochemistry (IHC) demonstrated reduced cytoplasmic *PCDH10* expression in breast tumor samples compared with normal tissues (Fig. 1E). Bioinformatics validation using the GENT2 database confirmed reduced *PCDH10* expression in breast cancer tissues compared to normal controls (Fig. 1F), and survival analysis revealed a better prognosis for patients with higher *PCDH10* expression (Fig. 1G). Analysis of TCGA data further revealed an association of higher *PCDH10* expression levels with ER-positive status (Table 2). Finally, MethHC database analysis corroborated our findings of increased *PCDH10* promoter methylation in breast cancer tissues versus normal tissues (Fig. 1H).

### Restoration of *PCDH10* suppresses breast tumor cell growth

**Figure 2:**
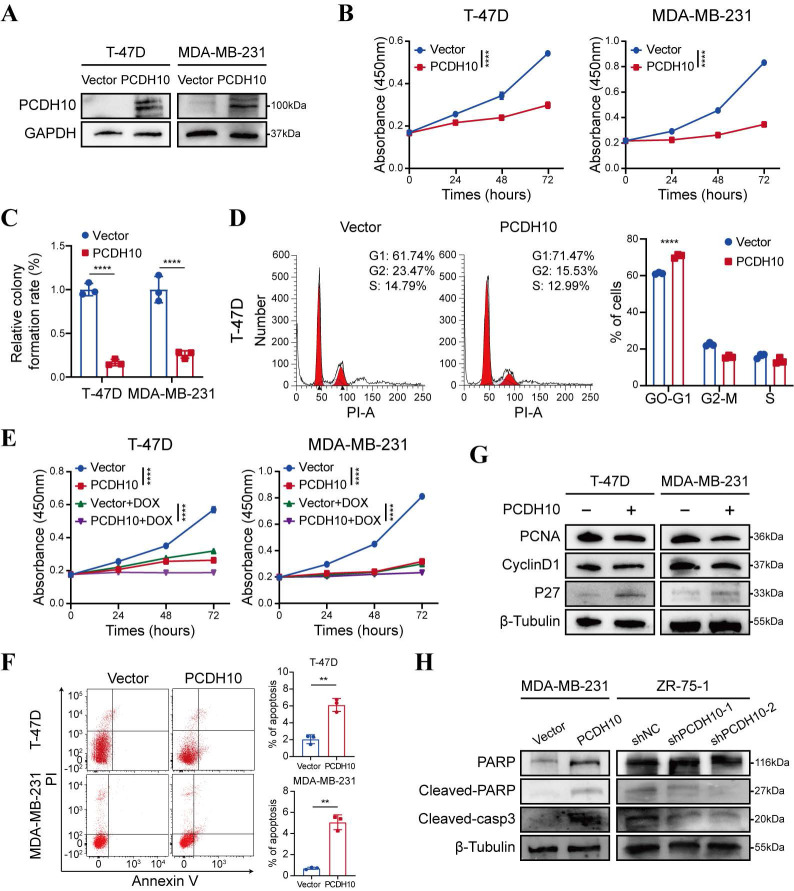
***PCDH10* expression inhibits the growth of breast cancer cells. A** WB detecting ectopic expression of PCDH10 in breast cancer cells line T-47D and MDA-MB-231. **B** CCK8 determining proliferation of T-47D and MDA-MB-231 cells in vector versus *PCDH10*-overexpressing (*PCDH10*-OE) group (n=3, two-way ANOVA). **C** Statistical analysis of colony formation of T-47D and MDA-MB-231 cells in vector versus *PCDH10*-OE group (n=3, two-way ANOVA). **D** Representative figures of cell cycle examined by FC (left) and statistical analysis of cell cycle (right) of T-47D cells in vector versus *PCDH10*-OE group (n=3, two-way ANOVA). **E** CCK8 determining effects of DOXO on breast cancer proliferation of T-47D and MDA-MB-231 cells in vector versus *PCDH10*-OE group (n=3, two-way ANOVA). **F** Representative plots of apoptosis assay determined by FC (left) and statistics analysis (right) of T-47D and MDA-MB-231 cells in vector versus *PCDH10*-OE group (n=3, t test). **G** Western blot analysis of PCNA, CyclinD1 and p27 in vector versus *PCDH10*-OE T-47D and MDA-MB-231 cells. **H** Western blot analysis of PARP, Cleaved-PARP, and Cleaved-casp3 in vector versus *PCDH10*-OE MDA-MB-231 cells and shNC versus shPCDH10 ZR-75-1 cells. Data are presented as mean ± SD; Each dot represents one sample; *p < 0.05. **p < 0.01. ***p < 0.001.****p < 0.0001. CCK8: Cell Counting Kit-8. FC: flow cytometry. NC, negative control. RT-PCR, semi-quantitative (RT)-PCR. WB, Western Blot. DOXO, Doxorubicin.

Promoter methylation-regulated disruption of *PCDH10* in breast cancer tissues and lack of this silencing in normal breast tissues suggested that *PCDH10* may be a functional TSG in breast cancer. A mammalian expression vector encoding *PCDH10* was transfected into breast cancer cells to further explore the effects of *PCDH10* on tumor biological functions. Based on RT-PCR results, both T-47D and MDA-MB-231 showed loss of *PCDH10* expression (Fig. 1A). These two cell lines were selected for constructing cells with stably expressed *PCDH10*. T-47D and MDA-MB-231 cells were transfected with vector or *PCDH10* plasmid, and transfection efficiency was further examined by Western blot (WB) (Fig. 2A). Proliferation of breast tumor cells was significantly suppressed by ectopic expression of *PCDH10* (Fig. 2B). Monolayer colony formation assays were applied to determine their colony formation abilities. *PCDH10* expression significantly reduced the colony formation of T-47D and MDA-MB-231 cells (Fig. 2C, Fig. S1A). To investigate the mechanism underlying the growth-suppressive effect of *PCDH10*, cell cycle and apoptosis assays were performed by flow cytometry (FC). The results demonstrated that *PCDH10* restoration increased cells in G0/G1 phase (Fig. 2D, Fig. S1B-C), enhanced their sensitivity to doxorubicin (Fig. 2E), and promoted baseline apoptosis (Fig. 2F). Furthermore, WB analyses suggested that *PCDH10* expression downregulated PCNA and cyclin D1 19, and upregulated p27 expression 20 (Fig. 2G). Therefore, *PCDH10* may contribute to growth inhibition and cell cycle arrest through associated pathways. Consistent with its pro-apoptotic role, *PCDH10* restoration increased cleaved caspase-3 levels and cleaved-PARP in MDA-MB-231, whereas *PCDH10* knockdown in ZR-75-1 attenuated apoptosis, decreasing both cleaved caspase-3 and cleaved-PARP levels (Fig. 2H). Collectively, these data establish *PCDH10* as a growth-suppressing tumor suppressor in breast cancer *in vitro.*

### Ectopic expression of *PCDH10* inhibits breast tumorigenesis and metastasis by suppressing EMT

**Figure 3:**
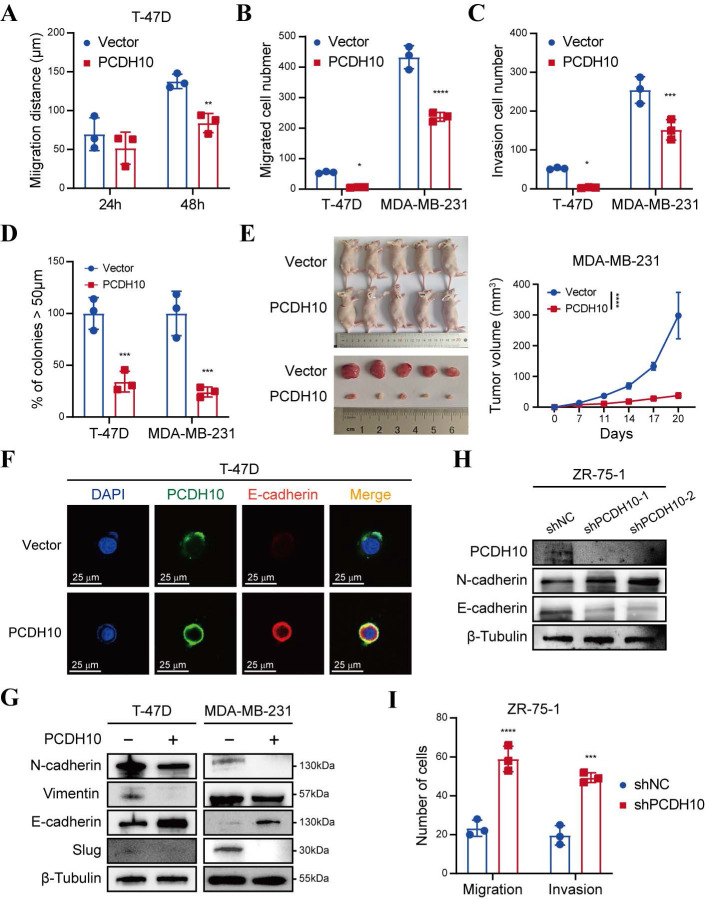
***PCDH10* expression suppresses metastasis *in vitro* and inhibits tumorigenesis *in vivo.* A-C** Statistical analysis of wound healing assay (A), migration assay (B) and invasion assay (C) of T-47D and MDA-MB-231 cells in vector versus *PCDH10*-OE group (n=3, two-way ANOVA). **D** Cell spheroid-forming assay determining effect of PCDH10 on stemness potential of T-47D and MDA-MB-231 cells (n=3, two-way ANOVA). **E** MDA-MB-231 tumor growth in nude mice implanted with vector versus *PCDH10*-OE cells (n=5, two-way ANOVA). All the mice harvested on day 20 with the tumors arranged in volume order. **F** IF staining showed representative images of PCDH10 (green) and E-cadherin (red) in T-47D cells. **G** Western blot analysis of N-cadherin, Vimentin, E-cadherin, and Slug in vector versus *PCDH10*-OE T-47D and MDA-MB-231 cells. **H** Western blot analysis of PCDH10, N-cadherin and E-cadherin in shNC versus shPCDH10 ZR-75-1 cells. **I** Statistical analysis of migration and invasion assay in shNC versus shPCDH10 ZR-75-1 cells (n=3, two-way ANOVA). Data are presented as mean ± SD; Each dot represents one sample. *p < 0.05. **p < 0.01. ***p < 0.001.****p < 0.0001.

To delineate the anti-metastatic function of *PCDH10*, we performed wound healing and Transwell® assays. Notably, *PCDH10*-expressing cells exhibited significantly delayed wound closure, compared to control groups (Fig. 3A, Fig. S2A-B). Consistent with this, Transwell® assays revealed significant suppression of migratory and invasive capacities, respectively (Fig. 3B-C, Fig. S2C). Spheroid-forming assay was performed to determine stemness potential, and results showed that *PCDH10* overexpression lowered the spheroid-forming rates of breast tumor cells (Fig. 3D, Fig. S3A). *In vivo*, *PCDH10* expression inhibited breast tumor development by reducing both tumor volume and weight (Fig. 3E, Fig. S3B). Epithelial-mesenchymal transition (EMT) plays a crucial role in tumor formation and metastasis 21-22. The hallmark features of EMT include loss of E-cadherin and gain of N-cadherin and Vimentin, which endow tumor cells with enhanced invasiveness and metastatic potential. To investigate the role of *PCDH10* in this process, we examined the expression of these markers in mouse tumor tissues. Immunohistochemistry (IHC) revealed that *PCDH10* restoration was associated with decreased N-cadherin but increased E-cadherin levels, which correlated with reduced tumor invasiveness and metastatic capacity (Fig. S3C). These findings were further confirmed by immunofluorescence (IF) staining of tumor cells (Fig. 3F, Fig. S3D). Subsequently, we performed WB analysis to detect EMT-related markers. Consistent with IHC results, expression of mesenchymal markers N-cadherin and Vimentin was downregulated in the *PCDH10-*restored group, whereas epithelial marker E-cadherin was upregulated (Fig. 3G). Additionally, expression of slug, a known EMT-inducing transcription factor, was also decreased (Fig. 3G). In contrast, in ZR-75-1, *PCDH10* knockdown led to increased N-cadherin and decreased E-cadherin expression, accompanied by enhanced cell migration and invasion (Fig. 3H, I, Fig. S3E). These results demonstrate that *PCDH10* inhibits tumor progression by suppressing the EMT process.

### *PCDH10* inhibits the Wnt/β-catenin pathway by modulating negative feedback factors and key components

**Figure 4:**
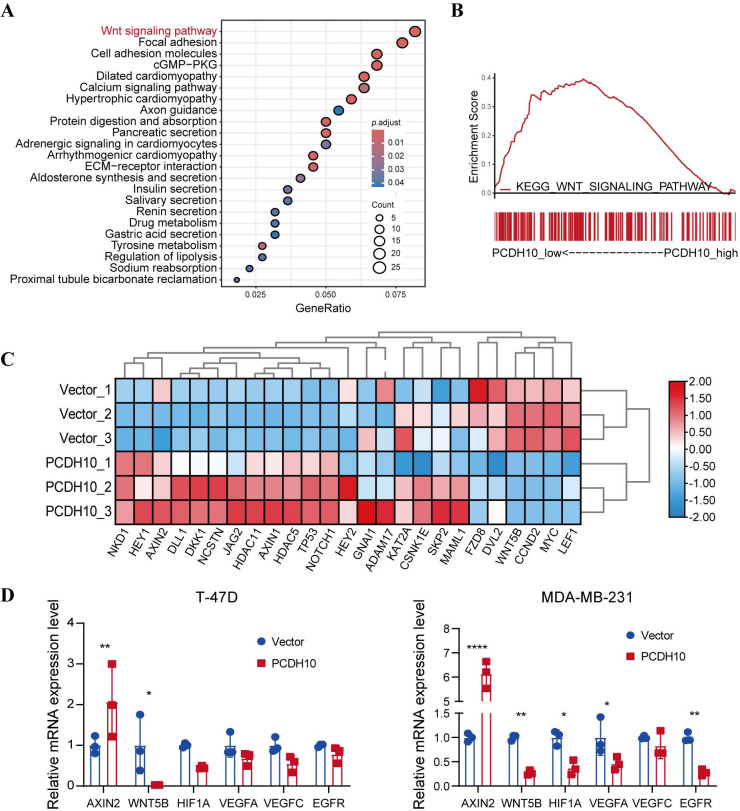
** Ectopic *PCDH10* expression inhibits EMT and downregulates multiple oncogenes in breast cancer cells. A** KEGG analysis of mRNA-seq data showing the enriched signaling pathways in MDA-MB-231 from vector groups, in comparison to those from *PCDH10*-OE groups. **B** GSEA enrichment analysis based on mRNA-seq data in MDA-MB-231 from vector groups, in comparison to those from *PCDH10*-OE groups. **C** Heatmap of β-catenin signaling pathway-associated genes based on mRNA sequencing data. **D** qRT-PCR exploring PCDH10 effects on *AXIN2*, *WNT5B*, *HIF1A*, *VEGFA*, *VEGFC*, *EGFR* expression in both T-47D and MDA-MB-231 (n=3, two-way ANOVA). Data are presented as mean ± SD; Each dot represents one sample. *p < 0.05. **p < 0.01. ***p < 0.001.****p < 0.0001.

To further elucidate the mechanisms underlying *PCDH10*-mediated tumor suppression, we conducted RNA sequencing after *PCDH10* restoration. KEGG analysis revealed that *PCDH10* perturbation is associated with multiple cancer-related signaling pathways, including Wnt/β-catenin (Fig. 4A). Confirming this association and extending prior findings linking *PCDH10* to Wnt signaling 23, 24, GSEA indicated that high *PCDH10* expression downregulates Wnt/β-catenin pathway (Fig. 4B). Therefore, we focused on Wnt/β-catenin pathway-related target genes from our RNA sequencing data. Results showed that *PCDH10* restoration upregulated the expression of Wnt/β-catenin negative regulators such as NKD1, AXIN2, and DLL1, while downregulating the expression of Wnt receptor FZD8 and transcriptional activator LEF1 (Fig. 4C). Subsequent qRT-PCR analysis validated the effects of *PCDH10* on the expression of these genes associated with Wnt/β-catenin pathway. Specifically, the expression of *AXIN2* was upregulated, while the expression of *WNT5B* 25, *HIF1A* 26, *VEGFA*, *VEGFC* 27, and *EGFR* 28 were downregulated (Fig. 4D). These results indicate that *PCDH10* inhibits the Wnt/β-catenin signaling pathway by upregulating negative regulators and downregulating key activators.

### *PCDH10* negatively regulates Wnt/β-catenin signaling via GSK-3β/β-catenin axis and protein-protein interactions

**Figure 5:**
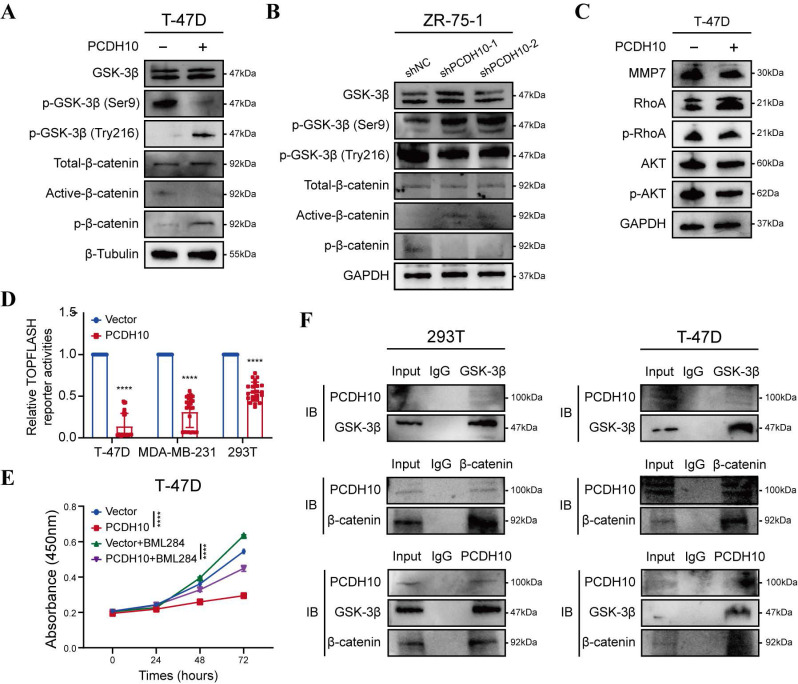
***PCDH10* expression antagonizes aberrant oncogenic β-catenin activation, likely through protein-protein interaction. A-B** Western blot analysis of GSK-3β and β-catenin status in vector versus *PCDH10*-OE of T-47D (A), or shNC versus shPCDH10 of ZR-75-1 (B). **C** Western blot analysis of MMP7, p-RhoA, and p-AKT in vector versus *PCDH10*-OE of T-47D. **D** *PCDH10* effect on TOPFLASH TCF-reporter construct activity was determined in T-47D, MDA-MB-231 and 293T (n = 24). **E** CCK8 was applied to detect inhibitive effect of *PCDH10* on Wnt/β-catenin pathway, and BML-284 was applied to activate β-catenin signaling in T-47D (n=3, two-way ANOVA). **F** Co-IP isolated extracts were applied for IB to confirm binding of PCDH10, GSK-3β and β-catenin in 293T and T-47D. Data are presented as mean ± SD; Each dot represents one sample. *p < 0.05. **p < 0.01. ***p < 0.001.****p < 0.0001. IB: Immunoblot.

To elucidate the effects of *PCDH10* on Wnt/β-catenin signaling pathway, we performed RNA-seq and qRT-PCR analyses and further validated the results by WB. Compared to control group, *PCDH10*-restorated T-47D and MDA-MB-231 cells exhibited significantly reduced levels of dephosphorylated (active) β-catenin and increased levels of phosphorylated β-catenin (inactive form targeted for degradation) (Fig. 5A, Fig. S4A). Dephosphorylated β-catenin primarily functions as a transcriptional co-activator in the nucleus. These findings support the hypothesis that PCDH10 inhibits the activation/stabilization of β-catenin. GSK-3β phosphorylation at Ser9 inhibits its kinase activity, stabilizing β-catenin, whereas phosphorylation at Tyr216 is required for its activity, promoting β-catenin phosphorylation and degradation. Our results showed that PCDH10 differentially regulated GSK-3β phosphorylation at these key sites to promote its activity. In *PCDH10*-overexpressing T-47D and MDA-MB-231 cells, phosphorylation at the inhibitory Ser9 site was reduced, while phosphorylation at the activating Tyr216 site was upregulated. This combination enhances GSK-3β kinase activity, thereby promoting β-catenin degradation (Fig. 5A, Fig. S4A). Conversely, in ZR-75-1 cells with *PCDH10* knockdown, phosphorylation at Tyr216 was reduced, and phosphorylation at Ser9 was increased. This dual change further suppresses GSK-3β activity, leading to the accumulation and activation of β-catenin (Fig. 5B).

Additionally, expression of MMP7, a downstream target gene of β-catenin, was examined to further confirm the inhibitory effects of *PCDH10* on the Wnt/β-catenin pathway. WB analysis revealed that *PCDH10* downregulated MMP7 expression in T-47D and MDA-MB-231 cells (Fig. 5C and Fig. S4B). PCDH10 also downregulated the levels of p-AKT and p-RhoA (Fig. 5C and Fig. S4B), both are known regulators of GSK-3β activity and β-catenin stability. Thus, PCDH10 may inhibit β-catenin activity partially by suppressing these signaling molecules.

To further validate the inhibitory effects of *PCDH10* on Wnt/β-catenin pathway, we employed a TOP-Flash/FOP-Flash TCF luciferase reporter assay. Luciferase activity was significantly reduced in cells expressing *PCDH10*, including 293T, T-47D, and MDA-MB-231 (Fig. 5D). Treatment with Wnt/β-catenin signaling activator BML-284 in cells expression *PCDH10* further confirmed its inhibitory effects (Fig. 5E, Fig. S4C). Finally, co-immunoprecipitation (Co-IP) followed by immunoblotting (IB) analyses confirmed direct protein-protein interactions among PCDH10, GSK-3β, and β-catenin (Fig. 5F, Fig. S4D). These results demonstrated that PCDH10 negatively regulates Wnt/β-catenin signaling through the GSK-3β/β-catenin axis via protein-protein interactions.

### PCDH10 upregulates LMNA expression through Akt signal pathway

**Figure 6:**
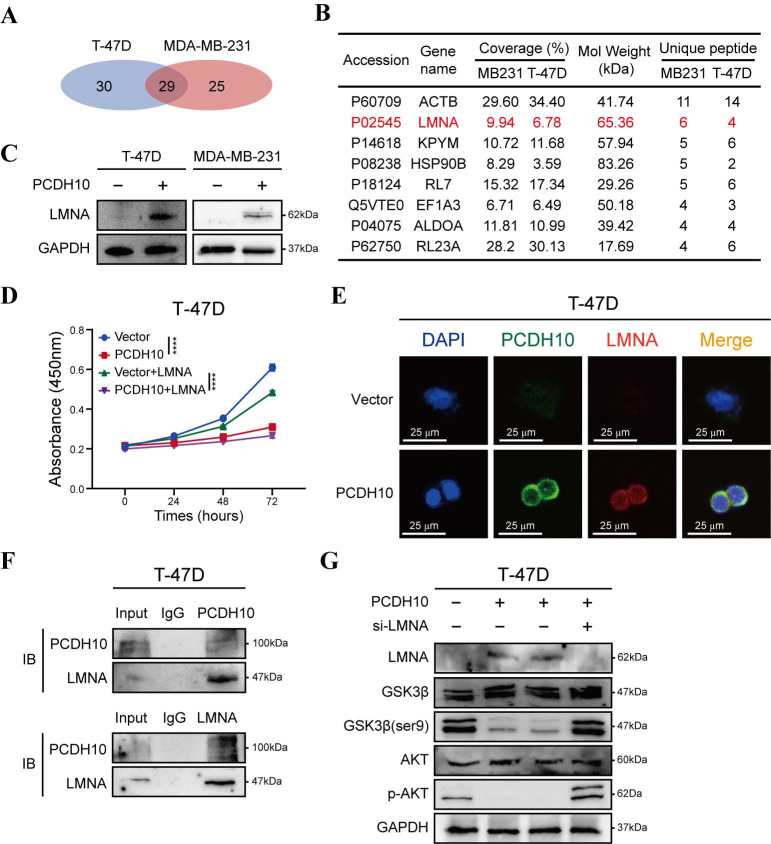
***PCDH10* upregulates LMNA expression through protein-protein interaction. A** Workflow of IP screening. **B** Distinct protein bands in the GEL were subjected to mass spectrometry, and the top eight interacting partners are shown. **C** Western blot analysis of LMNA in vector versus *PCDH10*-OE T-47D and MDA-MB-231 cells. **D** CCK8 determining LMNA effect on tumor cells growth in vector versus *PCDH10*-OE T-47D cells (n=3, two-way ANOVA). **E** IF staining showed representative images of PCDH10 (green) and LMNA (red) in vector versus *PCDH10*-OE T-47D cells. **F** IB and Co-IP performing to confirm protein-protein combination in T-47D. **G** Western blot analysis of LMNA, GSK-3β, GSK-3β (ser9), p-AKT and AKT in vector versus *PCDH10*-OE T-47D cells. IB: Immunoblot; WB: Western Blot. ****p < 0.0001.

To further elucidate the tumor-suppressive mechanisms of *PCDH10* in breast cancer, we employed co-immunoprecipitation (Co-IP) to identify PCDH10-interacting proteins, followed by mass spectrometry (MS) for protein identification. Silver staining of SDS-PAGE gels was used to visualize potential binding partners (Fig. S5A). Among 29 candidate binding proteins identified in *PCDH10*-expressing T-47D and MDA-MB-231 cells (Fig. 6A), we selected LMNA (Lamin A/C) for further investigation based on our experimental results (Fig. 6B). LMNA is a major component of the nuclear lamina, responsible for maintaining nuclear structure and function, and plays a crucial role in cell differentiation, gene regulation and cell cycle control.

Our results demonstrated that *PCDH10* expression upregulated LMNA expression (Fig. 6C). Additionally, functional assays revealed that LMNA mediated the growth-inhibitory effects of *PCDH10* (Fig. 6D, Fig. S5B). IF analysis further confirmed the co-localization of PCDH10 and LMNA in T-47D and MDA-MB-231 cells (Fig. 6E, Fig. S5C). Co-IP experiments showed direct protein-protein interaction between LMNA and PCDH10 (Fig. 6F, Fig. S5D).

Given the regulatory role of *PCDH10* in Akt/β-catenin signaling, we explored the impact of LMNA on the Akt pathway. Previous results indicated that *PCDH10* expression inhibits Akt signaling (Fig. 5C, Fig. S4B). In *PCDH10*-expressing T-47D and MDA-MB-231 cells, knockdown of LMNA partially restored p-Akt levels and increased p-GSK3β-Ser9, indicating LMNA mediates *PCDH10*'s inhibition of Akt signaling (Fig. 6G, Fig. S5E). Thus, *PCDH10* upregulates LMNA expression through protein-protein interactions, leading to inhibition of Akt signaling (via reduced p-Akt) and enhancement of GSK3β activity (via reduced p-Ser9) which can explain its anti-tumor effects.

## Discussion

Carcinogenesis is a multi-step process that involves the accumulation of multiple epigenetic and genetic alterations of oncogenes and TSGs 3. Reprograming the epigenetic landscape of the cancer genome is a promising therapeutic strategy 29. TSG methylation contributes to the pathogenesis of multiple cancers, including breast cancer 30, 31. The regulatory mechanism underlying the methylation system comprises several components: DNA methyltransferases and methyl-CpG binding proteins (MeCPs). DNA methyltransferases, including DNMT1, DNMT3A, DNMT3B (and DNMT2, though its primary role is debated 32), are involved in establishing and maintaining methylation patterns 33. MeCPs recognize methylated CpG sites. Key MeCPs members such as MeCP2, MBD1, MBD2 and MBD4 contain methylated DNA-binding domains (MBDs) 34. Additionally, histone modifications interact closely with DNA methylation in gene silencing 35.

We discovered the downregulation or silencing of *PCDH10* in most breast cancer tissues and cell lines tested. Methylation analysis and demethylation treatment indicated that the principal regulatory mechanism underlying *PCDH10* inactivation is aberrant promoter CpG methylation. RT-PCR also detected unmethylated alleles in YCC-B3 and SK-BR-3, suggesting that other repression regulatory mechanisms, such as histone modifications 36 might also contribute to its silencing. CpG methylation, which leads to the loss of TSG function, is closely associated with the onset and progression of multiple types of cancers 4. We observed *PCDH10* methylation in 83% of primary breast tumor tissues, whereas no *PCDH10* methylation was observed in adjacent non-tumor tissues. These results suggested that aberrant promoter methylation of *PCDH10* occurs early in the multistep process of breast carcinogenesis. Notably, *PCDH10* methylation is documented in other primary tumors, including esophageal 17, gastric 36, cervical 14 and hepatocellular cancers 37, reinforcing its broad tumor-suppressive role. While its timing relative to tumor grade or stage warrants further study, future work should validate *PCDH10* methylation in serum or tumor tissues as a diagnostic/screening biomarker.

This study establishes *PCDH10* methylation as a prognostic biomarker in breast cancer, with higher expression correlating with significantly longer patient survival and ER-positive status. The tumor-suppressive effects of *PCDH10* in breast cancer cells were determined using CCK8, wound healing, Transwell®, cell cycle, apoptosis, and cell spheroid formation assays *in vitro*, as well as subcutaneous tumor model *in vivo*. WB results further confirmed that *PCDH10* inhibited tumor cells growth, EMT and stemness. *PCDH10* also promoted G0/G1 phase arrest and apoptosis. Collectively, *PCDH10* restoration coordinately suppresses breast cancer growth, metastasis, and stem-like properties, highlighting its therapeutic potential.

EMT, the process whereby epithelial cells transdifferentiate into motile mesenchymal cells, is critical for metastatic progression in breast cancer. In our study, restoration of *PCDH10* significantly inhibited the expression of EMT and stemness markers in breast cancer cells, including N-cadherin, Vimentin, Slug, among others. RNA-seq KEGG analysis revealed enrichment in Wnt signaling pathway, focal adhesion, cell adhesion molecules, and others. Mechanistically, *PCDH10* downregulated Wnt/β-catenin target genes Axin2 and cyclin D1 38. Furthermore, *PCDH10* downregulated multiple β-catenin/TCF-LEF transcriptionally regulated genes and related oncogenes, including *WNT5B, HIF1A, VEGFA, VEGFC and EGFR*. *WNT5B* 39 is closely related to Wnt/β-catenin pathway and have been reported to promote the metastatic ability of cancer cells. HIF1A exerts tumor-promoted effects through regulating Wnt/β-catenin signaling 26. *VEGFA* and* VEGFC* 27 are also regulated by Wnt/β-catenin signaling. Convergence exists between Wnt/β-catenin and EGFR signaling 28. These results confirmed the tumor-suppressive function of *PCDH10* through potent inhibition of Wnt/β-catenin pathway.

Protocadherins mediate cell sorting, selective cell-cell adhesion, and homophilic binding 40. Although we demonstrate *PCDH10* is predominantly located in the cytoplasm, the functions of protocadherins can change during carcinogenesis, and they can act as signaling molecules that participates in regulating signaling pathways 41.

WNTs are glycoproteins secreted into the extracellular matrix. The canonical Wnt/β-catenin pathway features “Wnt on” and “Wnt off” states; the presence of WNT ligands “turn on” the pathway by promoting the accumulation of active β-catenin through inactivation of GSK-3β 42. GSK-3β, which forms a complex with CK1α and APC to target β-catenin for phosphorylation and subsequent proteasomal degradation 43. We demonstrate that *PCDH10* enhances the active form of GSK-3β, suppressing β-catenin stabilization. Consistently, *PCDH10* overexpression significantly reduced TOPFLASH reporter activity. Since TCF/Lef1 (T cell factor/lymphoid enhancer factor-1) mediates canonical WNT-triggered gene transcription, this supports the finding that *PCDH10* antagonized Wnt/β-catenin signaling by downregulating active β-catenin levels. Additionally, *PCDH10* suppressed MMP7 expression, a β-catenin downstream target gene. Furthermore, *PCDH10* suppressed p-AKT and p-RhoA expression, which are critical regulators of β-catenin 44. Critically, co-immunoprecipitation verified direct binding between PCDH10, GSK-3β and β-catenin, establishing PCDH10 as a scaffold protein that orchestrates β-catenin degradation through GSK-3β activation while concurrently inhibiting AKT/RhoA-mediated β-catenin stabilization. Future studies employing specific pathway modulators (e.g., Wnt activators/inhibitors, PI3K/Akt inhibitors) will be valuable to further delineate the pathway dependencies and consolidate these mechanistic insights.

We further established that LMNA, a nuclear intermediate filament, directly interacts with PCDH10. *PCDH10* overexpression significantly increased LMNA protein levels in breast cancer cells. Notably, LMNA functions as a TSG in carcinomas 45, with its loss of expression correlating with poor prognosis and shorter survival in breast cancer patients 46. Consistent with this role, our functional studies indicated that LMNA is a critical downstream mediator of *PCDH10*'s tumor-suppressive function. Emerin, an inner nuclear membrane protein, requires LMNA for its proper localization 47 and interacts with β-catenin, restricting its nucleus access 48. The upregulation of LMNA by *PCDH10* might inhibit β-catenin activity by promoting emerin-mediated sequestration, though this model requires further experimental validation.

**Figure 7:**
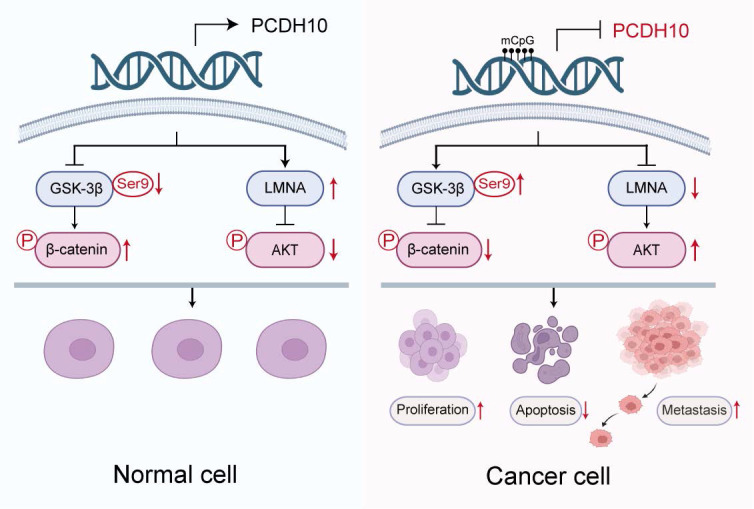
** Schematic illustration of the tumor-suppressive mechanism of *PCDH10* in breast cancer.** In breast cancer cells, promoter methylation silences *PCDH10*, resulting in increased GSK-3β (Ser9) phosphorylation, decreased β-catenin phosphorylation, downregulation of LMNA, and activation of Akt signaling. These changes collectively promote tumor cell proliferation, inhibit apoptosis, and enhance metastasis.

In summary, this study reveals frequent epigenetic silencing of the tumor suppressor *PCDH10* in breast cancer, which correlates with ER-positive status and longer patient survival. Mechanistic analyses indicated that *PCDH10* antagonizes Wnt/β-catenin signaling through its interaction with GSK-3β/β-catenin complex as a scaffold protein and *via* LMNA upregulation. *PCDH10* additionally suppresses AKT and RhoA phosphorylation (Fig. 7). The frequent aberrant epigenetic silencing event likely plays an essential role in breast cancer carcinogenesis, also positioning *PCDH10* methylation as a promising diagnostic or prognostic biomarker.

## Materials and Methods

### Tumor samples, normal tissues and cell lines

RNA samples from human normal adult breast tissue were commercially obtained (BioChain Institute, Hayward, CA and Millipore Chemicon, Billerica, MA; or Stratagene, La Jolla, CA). Primary Breast carcinoma, adjacent non-cancerous tissues, and normal breast tissues were collected from the First Affiliated Hospital of Chongqing Medical University (CQMU) as previously described 9, 49. Breast cancer cell lines (MDA-MB-231, T-47D, MCF7, BT549, MDA-MB-468, SK-BR-3, ZR-75-1, YCCB1, and YCCB3) and 293T were used. Cells were obtained from collaborators or purchased from American Type Culture Collection (ATCC, Manassas, VA). Cells were routinely maintained in DMEM or RPMI-1640 medium (Gibco) supplemented 10% fetal bovine serum (FBS, Gibco).

### DNA and RNA extraction

Total RNA and DNA were extracted from tissues and cells using TRIzol® Reagent (Invitrogen, Carlsbad, CA) according to the manufacturer's protocol. For DNA extraction from normal and breast tumor tissues, samples were homogenized using liquid nitrogen and incubated in a solution containing 200 µg/ml proteinase K, 50 mM EDTA, 2% N-lauryl-sarcosyl, 10 mM Tris-HCl (pH 8.0), and 10 mM NaCl for 20 h at 55ºC. The samples were then extracted with phenol-chloroform extraction and subjected to ethanol precipitation. For RNA-seq analysis, cells were lysed with TRIzol® Reagent, and data analysis were performed by LC Sciences (Hangzhou, China).

### Promoter methylation analysis and bisulfite treatment

Methylation-specific PCR (MSP) 49, bisulfite modification of DNA, and BGS 50 were performed as described previously. The MSP primers had been tested previously, and direct sequencing was used to analyze the MSP products to confirm that the MSP system was specific. Primers used were listed in Supplementary table 1. For BGS, the following primers were used to amplify bisulfite-treated DNA: BGS1: 5′- GTT GAT GTA AAT AGG GGA ATT-3′ and BGS2: 5′-CTT CAA CCT CTA AAC CTA TAA-3′. The PCR products were cloned into the PCR4-Topo vector (Invitrogen). Randomly selected 8-10 colonies were applied for further sequencing.

### 5-aza-2'-deoxycytidine (Aza) and Trichostatin A (TSA) treatment

The demethylating agent Aza (Sigma-Aldrich, St Louis, MO, USA) and the histone deacetylase inhibitor TSA (Cayman Chemical Co., Ann Arbor, MI, USA) were used. For Aza and TSA treatment, cells were treated with Aza (10 µM, Sigma) for 3 days followed by TSA (100 ng/mL) for 1 day.

### Cloning PCDH10 and constructing the expression vector

AccuPrime Pfx DNA polymerase (Invitrogen) and a full-length clone of KIAA1400 (a kind gift from Kazusa DNA Research Institute, Chiba, Japan) were used to generate the PCR product and to construct the pcDNA3.1(+)-*PCDH10* plasmid. Sequencing was used to confirm all construct sequences and orientations.

### Construction of cells with stable *PCDH10* expression

MDA-MB-231 and T-47D were chosen to establish cell lines stably expressing *PCDH10*. Following the manufacturer's instructions, cells were transfected with the *PCDH10* plasmid using Opti-MEM (Invitrogen) and Lipofectamine 3000 (Invitrogen). Transfected MDA-MB-231 and T-47D were cultured for additional 48 hours and then selected with G418. The cell cultures used are mixed cultures of stable transfectants. WB were applied to confirm *PCDH10* ectopic expression.

### Reverse transcription-PCR, semi-quantitative (RT)-PCR and qRT-PCR

Reverse transcription of RNA was performed with GoScriptTM reverse transcriptase (Promega, Madison, WI), and reaction conditions were as previously reported. AmpliTaq Gold T (Applied Biosystems, Foster City, CA, USA) was used to perform Semi-quantitative (RT)-PCR as previously reported. Primers used are listed in Supplementary Table 1. Based on the instrument manual (HT7500 System; Applied Biosystems, Foster), qRT-PCR was performed using SYBR Green (Promega). The 2-∆Ct method was used to calculat relative expression. GAPDH was amplified as a control for RNA integrity.

### Proliferation assay

Cell proliferation was assayed using the CCK-8 (Cell Counting Kit-8, Beyotime, Shanghai, China) at 0, 24, 48 and 72 hours. In the 96-well plates, breast cancer cells with or without ectopic expression of *PCDH10* were seeded (2000 cells per well). A microplate reader was used to examine the absorbance at 450 nm (TECAN, Infinite M200 Pro).

### Agents

To detect sensitivity to doxorubicin (DOXO), cells were treated with DOXO (Abcam, ab120629) at 1 μg/mL for 24 hours, and then subjected to the proliferation assay. To investigate the inhibitory effect of *PCDH10* on Wnt/β-catenin signaling, cells were treated with 10 μM BML-284 (MCE, HY-19987) for 24 hours. Treated cells were then analyzed using the CCK-8 assay. Vector-transfected cells and DMSO-treated cells were used as controls.

### Colony formation assay

Cells expressing *PCDH10* or an empty vector, along with wild-type breast cancer cells (MDA-MB-231, 800 cells/well; T-47D, 1200 cells/well) were seeded in 6-well plates. Untransfected breast cancer cells were eliminated by G418 selection. Surviving colonies were counted (>50 cells/colony).

### Cell cycle and apoptosis analyses

Flow cytometry (FC) analysis was performed to assess apoptosis and cell cycle distribution. Cells (10 × 10^5^) were cultured in 6-well plates for 48 hours and subsequently harvested. Cells were processed as previously described 49. Cell cycle distribution and apoptosis were analyzed using a BD FACSCanto II Flow Cytometer; cell cycle data were analyzed using ModFit LT software and apoptosis data were analyzed using FlowJo software.

### Wound healing assays

The scratch wound assay was used to assess cell mobility in 6-well plates. Empty vector-transfected and *PCDH10*-expressing cells were cultured until confluent. Cells were washed with PBS and then cultured in serum-free RPMI-1640. After scratching the monolayer, images were acquired using a microscope (Olympus, Tokyo, Japan) and wound widths were measured.

### Transwell® assay

The invasive or migratory abilities of breast cancer cells (MDA-MB-231, 1 × 10^4^ cells/well, 48 hours; T-47D, 2×10^4^ cells/well, 72 hours) were determined using Transwell® plates coated with or without Matrigel (C0371, beyotime). Breast cancer cells were starved overnight and processed as described previously 31.

### Cell spheroid formation assay

Spheroid-forming assays were performed using *PCDH10* stably-expressing breast cancer cells as described previously 49. Vector-transfected cells were used as controls. Following continuous culture until distinct, compact tumor spheroids formed (containing > 50 cells per spheroid), the spheroids were visualized under an inverted phase-contrast microscope (Olympus, Tokyo, Japan) and subsequently counted.

### *In vivo* tumor model

Ten female nude mice were obtained commercially from Enswell Biotechnology Co., Ltd, China. Mice were randomly divided into two groups, and subcutaneously injected with MDA-MB-231 cells (2 × 10^6^ cells) stably expressing empty-vector or *PCDH10*. Body weight and tumor size were measured and recorded every 3 days. Mice were euthanized before the volume of any tumor reached 1 cm^3^. Excised tumors were photographed and fixed in formalin for paraffin embedding.

### IF and IHC

MDA-MB-231 and T-47D cells were transfected with empty vector or *PCDH10* plasmid. Transfected cells were cultured on glass coverslips in 6-well plates. After washing three times with PBS, samples were processed as previously reported 9. The primary antibodies used were as follows: E-cadherin (sc-21791, Santa Cruz, 1:100), LMNA (HA601274, HUABIO, 1:100), and PCDH10 (21859-1-AP, proteintech, 1:50). DAPI was used as a nuclear counterstain. Anti-rabbit IgG Alexa Fluor®488 (ab150077, Abcam, 1:200) and anti-mouse IgG Alexa Fluor® 594 (ab150116, Abcam, 1:200) were applied as secondary antibodies. Co-localization analysis was performed using using LAS AF software (Leica confocal microscope). For IHC, human patient and mouse samples were analyzed following a previously published protocol 51. The antibody used for detection was anti-PCDH10 (21859-1-AP, Proteintech, 1:200), E-cadherin (EM0502, HUABIO, 1:500), and N-cadherin (ET1607-37, HUABIO, 1:200).

### Western blot (WB) and Co-immunoprecipitation (Co-IP)

For WB, cells were lysed in ice-cold PBS containing 1 mM PMSF (Thermo Fisher) and cocktail (Thermo Fisher). Protein lysates were mixed with loading buffer and separated by SDS-PAGE as previously described 52. Membranes were incubated with the indicated monoclonal antibodies, including PCDH10 (21859-1-AP, Proteintech, 1:1000), PCNA (EM111201, HUABIO, 1:1000), CyclinD1 (ER0722, HUABIO, 1:1000), P27 (ET1608-61, HUABIO, 1:1000), PARP (9532, Cell Signaling Technology, 1:1000), Cleaved-PARP (HA722218, HUABIO, 1:1000), Cleaved-casp3 (25128-1-AP, Proteintech, 1:1000), N-cadherin (ET1607-37, HUABIO, 1:1000), Vimentin (ET1610-39, HUABIO, 1:1000), E-cadherin (ET1607-75, HUABIO, 1:1000), Slug (9585, Cell Signaling Technology, 1:1000), β-catenin (8480, Cell Signaling Technology, 1:1000), non-phospho (Active) β-catenin (8814, Cell Signaling Technology, 1:1000), p-β-catenin (HA721580, HUABIO, 1:1000), GSK-3β (ET1607-71, HUABIO, 1:1000), p-GSK-3β ser9 (ET1607-60, HUABIO, 1:1000), p-GSK-3β try216 (ET1607-54, HUABIO, 1:1000), LMNA (ET7110-12, HUABIO, 1:1000), AKT (10176-2-AP, Proteintech, 1:1000), p-AKT (4060, Cell Signaling Technology, 1:1000), MMP7 (ab207299, Abcam, 1:1000), RhoA (ET1611-10, HUABIO, 1:1000), p-RhoA (AF3352, Affinity, 1:1000), β-tubulin (2146, Cell Signaling Technology, 1:1000), GAPDH (2118, Cell Signaling Technology, 1:1000). Membranes were washed three times with PBST, then incubated with horseradish peroxidase (HRP)-conjugated anti-rabbit IgG (Biosharp, 1:2000) or anti-mouse IgG (Biosharp, 1:2000). Signals were detected using enhanced chemiluminescence (ECL) on a Gel Imager System (FX5, Vilber Lourmat).

For Co-IP, Protein A/G Magnetic Beads (HY-K0202, MCE) were used according to a published protocol (ref. 60). Lysates were incubated with antibodies against: Flag tag (DYKDDDDK, #14793, CST, 1:50), GSK-3β (sc-377213, Santa Cruz, 1:50), β-catenin (sc-65480, Santa Cruz, 1:50), and LMNA (sc-376248, Santa Cruz, 1:50). Co-IP complexes were analyzed by SDS-PAGE and WB. For detection, anti-mouse IgG (#A25012, Abbkine, 1:3000) was used. IP efficiency was confirmed by silver staining (P0017S, Beyotime). Entire gel lanes were excised for mass spectrometry (MS) analysis (Wuhan GeneCreate Biological Engineering Co., Ltd.).

### Luciferase activity assay

Cells were co-transfected with TOPFLASH (TCF reporter) and Renilla luciferase (internal control) constructs, along with empty vector or *PCDH10*, using Lipofectamine 3000. Cells were harvested 48 hours post-transfection. Lysates were transferred to a 96-well OptiPlate™, and luciferase activity was measured (TECAN Infinite M200 Pro). Firefly luciferase activity (TOPFLASH) was normalized to Renilla luciferase activity. Measurements were performed in triplicate 53.

### Bioinformatics and statistical analysis

*PCDH10* methylation in breast cancer was analyzed using the MethHC database. The results are presented as mean ± SD. Functional analysis, qRT-PCR, WB, and other assays were performed at least three times. Patient characteristics and methylation status were obtained from TCGA. Kaplan-Meier survival curves and GENT2 expression data accessed online. GraphPad Prism (version 8.0) was used for statistical analyses. For RNA-seq analysis, total RNA was extracted from MDA-MB-231 cells using Trizol reagent. Sequencing libraries were prepared and sequenced on the NovaSeq platform by LC-Bio Technologies (Hangzhou) Co., Ltd. Pathway enrichment was analyzed using the KEGG database with R clusterProfiler (v4.1.0), and GSEA (v4.0.3) was applied for further analysis. Statistical comparisons were conducted using t-test, two-way ANOVA, and log-rank test. *p* values <0.05 were considered to represent a statistically significant difference. * *p* < 0.05; ** *p* < 0.01; *** *p* < 0.001; **** *p* < 0.0001.

## Supplementary Material

Supplementary figures and table.

## Figures and Tables

**Table 1:** *PCDH10* promoter methylation status in primary breast tumors

Samples	*PCDH10* promoter		Frequency of methylation
	Methylation	Unmethylation	
BrCa (n=52)	43	9	83%
BA (n=5)	0	5	0%
BNP (n=14)	2	12	14%

Note: BA, breast cancer adjacent tissues; BrCa, breast cancer; BNP, breast normal tissues.

**Table 2:** Relationship between clinicopathological features and *PCDH10* expression in breast cancer patients (TCGA)

Clinicopathological features	Cases (n=600)	Low expression	High expression	X^2^	*p* value
*Age*				0.02	0.63
> 55	351	172(49.0%)	179 (51.0%)		
< 55	249	127(51.0%)	122(49.00%)		
*ER*				0.146	<0.001
Negative	134	85 (63.4%)	49 (36.6%)		
Positive	466	214(45.9%)	252 (54.1%)		
NULL=0					
*PR*				0.067	0.102
Negative	190	104(54.7%)	86 (45.3%)		
Positive	410	193(47.1%)	217 (52.9%)		
NULL=0				0.019	0.651
*HER2*					
Negative	500	252(50.4%)	248 (49.6%)		
Positive	92	44 (47.8%)	48 (52.2%)		
NULL=8					
*Metastasis*				0.036	0.374
M0	585	292(49.9%)	293 (50.1%)		
M1	11	4 (36.4%)	7 (63.6%)		
NULL=4					
*Stage (AJCC)*				0.007	0.876
I-II	447	224(50.11%)	223(49.89%)		
III-IV	144	71 (49.31%)	73 (50.69%)		
NULL=9					

Note. Null* Indeterminate / equivocal; PR: progesterone receptor; ER: estrogen receptor; HER2: Human epidermal growth factor receptor 2; AJCC: American Joint Committee on Cancer.

## Abbreviations

CCK8: cell counting kit-8
BGS: bisulfite genomic sequencing
DOXO: doxorubicin
Co-IP: co-immunoprecipitation
EMT: epithelial-mesenchymal transition
FC: flow cytometry
CGI: CpG islands
IB: immunoblot
IF: immunofluorescence
IHC: immunohistochemistry
MS: mass spectrum
MSP: methylation-specific PCR
RNA-seq: RNA sequencing
TSG: tumor suppressor gene
TCGA: The Cancer Genome Atlas
WB: western blot
