# Supplementary material for: Classic Protocadherin PCDH10 Functions as a Tumor Suppressive Scaffold Protein Antagonizing Oncogenic WNT/β-catenin Signaling in Breast Carcinogenesis
Source: Int J Biol Sci. 2026 Jan 8;22(3):1480–95. doi: 10.7150/ijbs.127857 (PMC12839071; doi:10.7150/ijbs.127857)
Supplement: Supplementary file 1 — Supplementary figures and table. [file ijbsv22p1480s1.pdf]

Protocadherin PCDH10 Functions as a Tumor Suppressive Scaffold Protein Antagonizing Oncogenic WNT/β-catenin Signaling in Breast Carcinogenesis

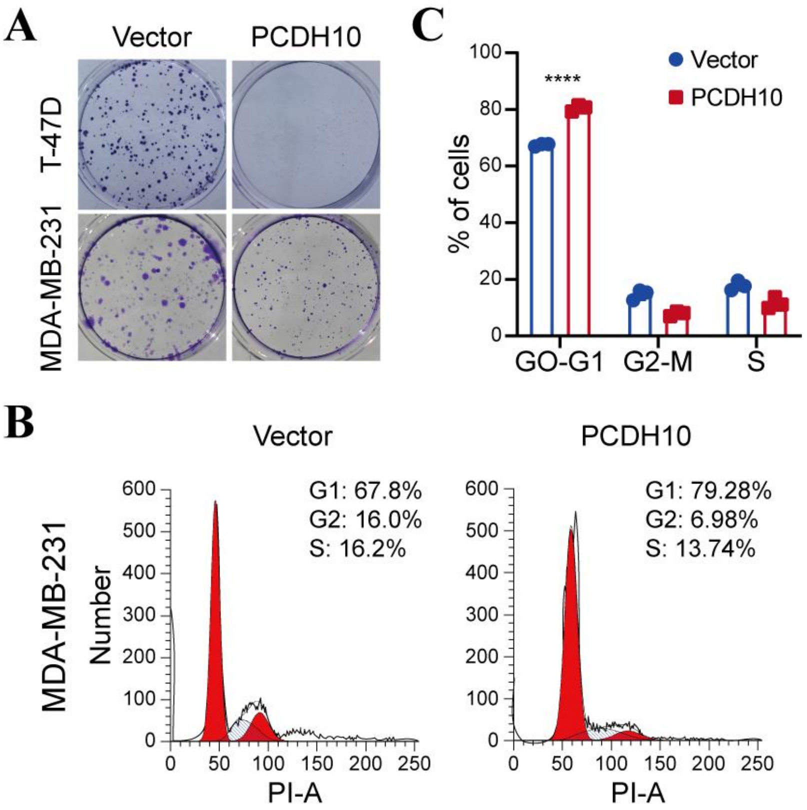

Figure S1. PCDH10 Regulates Proliferation and Apoptosis in Breast Cancer Cells. A Representative images of colony formation of T-47D and MDA-MB-231 cells in vector versus PCDH10-OE group (n=3, two-way ANOVA). **B-C** Representative figures of cell cycle examined by FC (B) and statistical analysis of cell cycle (C) of MDA-MB-231 cells in vector versus PCDH10-OE group (n=3, two-way ANOVA). ****p < 0.0001.

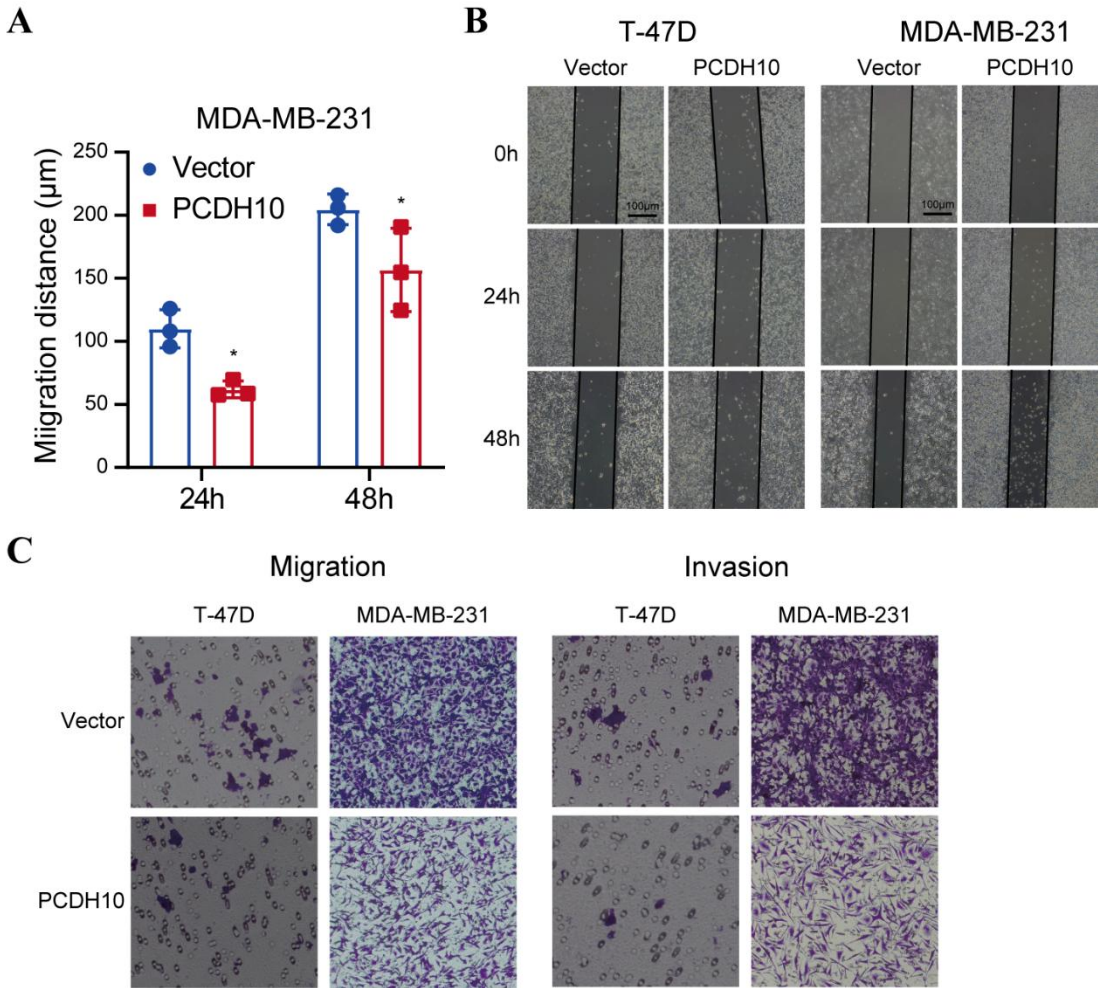

Figure S2. PCDH10 impairs the migratory and invasive capacities of breast cancer cells. A Statistical analysis of wound healing assay of MDA-MB-231 cells in vector versus PCDH10-OE group (n=3, two-way ANOVA). **B** Scratch assays indicating the effect of PCDH10 on motility of T-47D and MDA-MB-231 cells. **C** Transwell assays showing the effect of PCDH10 on migration and invasion of T-47D and MDA-MB-231. *$p < 0.05$.

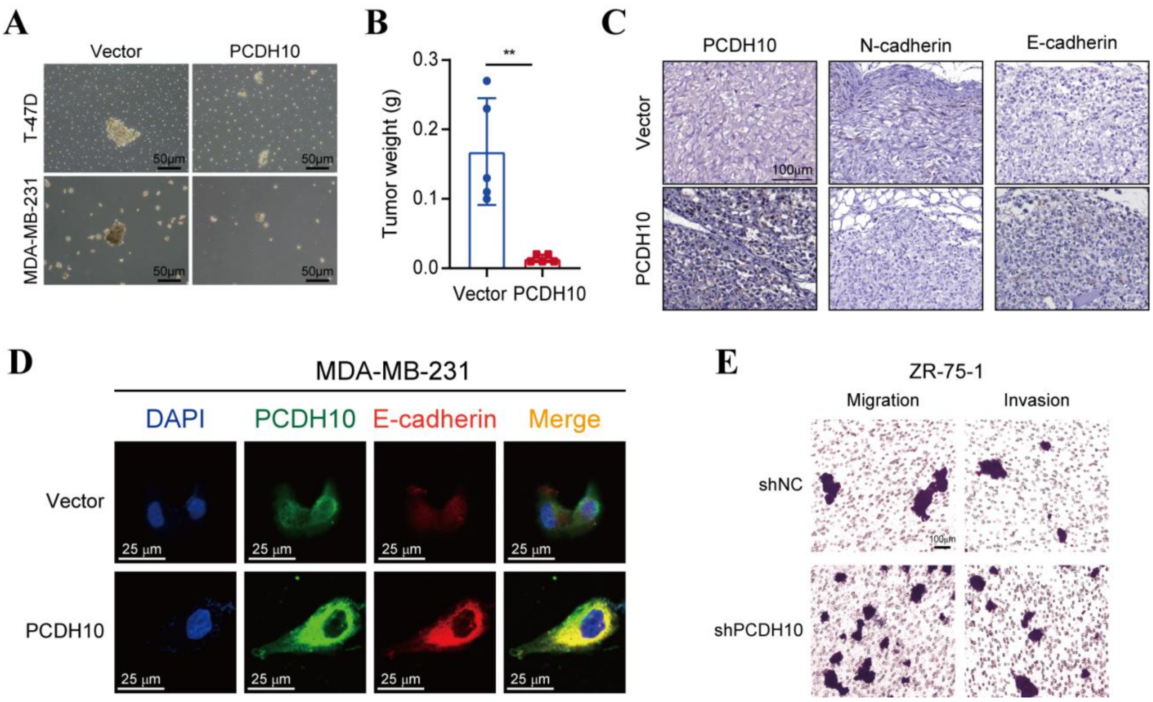

Figure S3. PCDH10 reverses EMT and reduces stem-like properties. A Representative images of cell spheroid-forming assay of T-47D and MDA-MB-231 cells in vector versus PCDH10-OE group (n=3, two-way ANOVA). **B** Tumor weights from nude mice xenografted with vector versus PCDH10-OE MDA-MB-231 cells (n=5, t test). **C** Immunohistochemical (IHC) staining of N-cadherin and E-cadherin in xenograft tumor sections derived from vector and PCDH10-OE MDA-MB-231 cells. **D** IF staining showed representative images of PCDH10 (green) and E-cadherin (red) in MDA-MB-231 cells. **E** Transwell assays showing the effect of PCDH10 on migration and invasion of ZR-75-1 cells. $^{**}p < 0.01$.

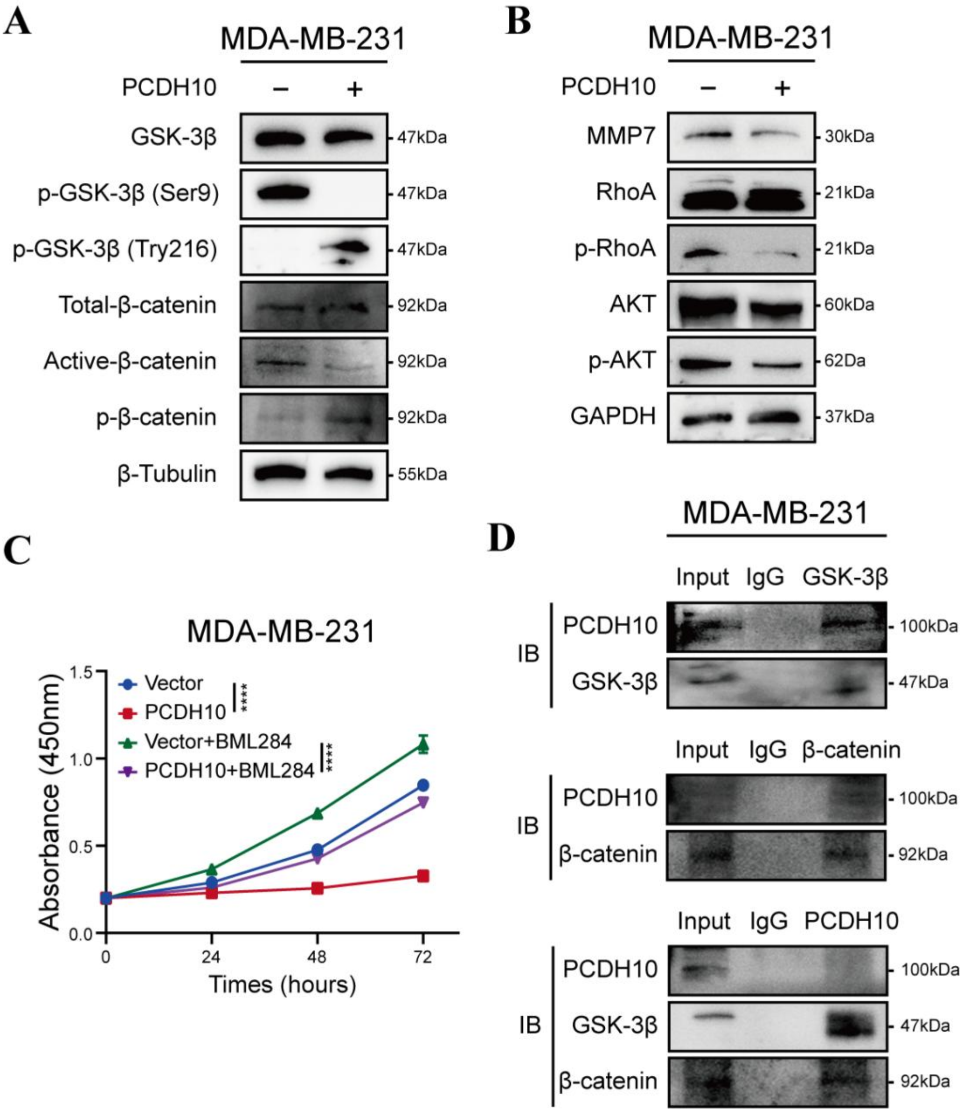

Figure S4. PCDH10 expression confirms the inhibition of Wnt/β-catenin signaling in MDA-MB-231 cells. A Western blot analysis of GSK-3β and β-catenin status in vector versus PCDH10-OE MDA-MB-231. **B** Western blot analysis of MMP7, p-RhoA, and p-AKT in vector versus PCDH10-OE of MDA-MB-231. **C** CCK8 was applied to detect inhibitive effect of PCDH10 on Wnt/β-catenin pathway, and BML-284 was applied to activate β-catenin signaling in MDA-MB-231 (n=3, two-way ANOVA). **D** Co-IP isolated extracts were applied for IB to confirm binding of PCDH10, GSK-3β and β-catenin in MDA-MB-231. ****p < 0.0001. IB: Immunoblot.

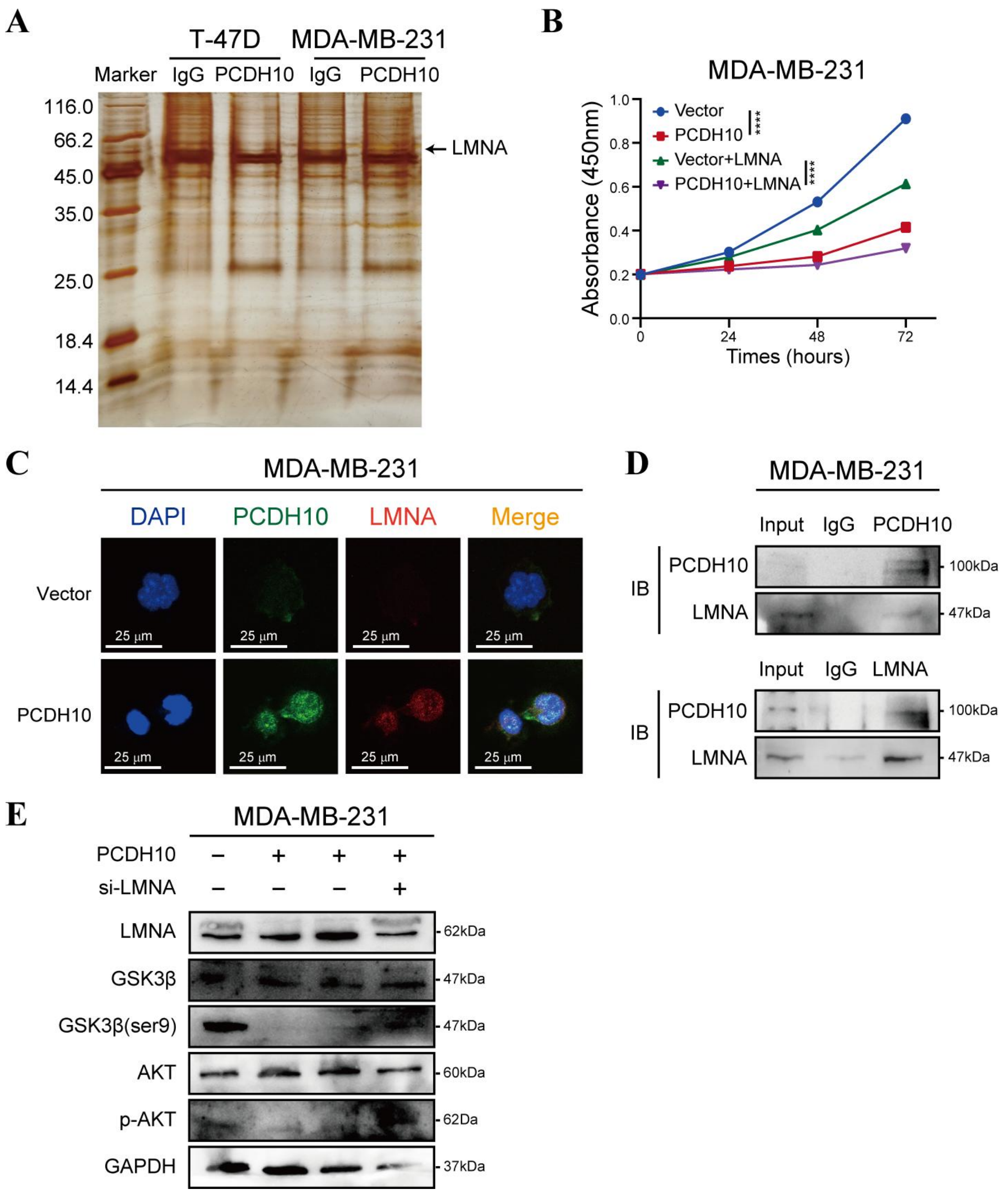

Figure S5. PCDH10 upregulates LMNA and inhibits Akt signaling in MDA-MB-231 cells. A Endogenous PCDH10 was immunoprecipitated with anti-PCDH10 antibody, and silver staining was performed to visualize the proteins interacting with PCDH10. **B** CCK8 determining LMNA effect on tumor cells growth in vector versus PCDH10-OE MDA-MB-231 cells (n=3, two-way ANOVA). **C** IF staining showed representative images of PCDH10 (green) and LMNA (red) in vector versus PCDH10-OE MDA-MB-231 cells. **D** IB and Co-IP performing to confirm protein-protein combination in MDA-MB-231. **E** Western

blot analysis of LMNA, GSK-3β, GSK-3β (ser9), p-AKT and AKT in vector versus PCDH10-OE MDA-MB-231 cells. IB: Immunoblot; WB: Western Blot. ****p < 0.0001.

Supplementary Table 1. List of PCR primers used in this study

PCR	Primer	Sequence (5'-3')	
RT-PCR / qRT-PCR	*PCDH10 F*	ACTGCTATCAGGTATGCCTG	**219bp**
	PCDH10 R	GTCTGTCAACTAGATAGCTG	
	Wnt5bF	AAATGCCACGGCGTCTCG	**163bp**
	Wnt5bR	GGGTGAAGCGGCTGTTGA	
	HIF1AF	ACCATCAGCTATTTGCGTGTGA	**143bp**
	HIF1AR	ATCATGTCACCATCATCTGTGAG	
	VEGFAF	CACACAGGATGGCTTGAAGA	**136bp**
	VEGFAR	AGGGCAGAATCATCACGAAG	
	VEGFCF	CTTGCTGGGCTTCTTCTC	**180bp**
	VEGFCR	GACCGTAACTGCTCCTCC	
	EGFRF	TTCCTATGCCTTAGCAGTCTTAT	**142bp**
	EGFRR	GATGCTCTCCACGTTGCACAG	
	Axin2F	*GCAGCTCAGCAAAAAGGGAAAT*	**114bp**
	Axin2R	*TACATGGGGAGCACTGTCTCGT*	
MSP	*PCDH10-bM1*	TCGTTAAATAGATACGTTACGC	**153bp**
	PCDH10-bM2	TAAAAACTAAAAACTTTCCGCG	
	PCDH10-bU1	GTTGTTAAATAGATATGTTATGT	**155bp**
	PCDH10-bU2	CTAAAAACTAAAAACTTTCCACA	

Note. qPCR: quantitative Polymerase Chain Reaction;
RT-PCR: Reverse Transcription-Polymerase Chain Reaction;
MSP: Methylation-specific PCR.
